# Comparison of Whole and Gutted Baltic Herring as a Raw Material for Restructured Fish Product Produced by High-Moisture Extrusion Cooking

**DOI:** 10.3390/foods9111541

**Published:** 2020-10-26

**Authors:** Anni Nisov, Heikki Aisala, Ulla Holopainen-Mantila, Hanna-Leena Alakomi, Emilia Nordlund, Kaisu Honkapää

**Affiliations:** VTT Technical Research Centre of Finland, Ltd., P.O. Box 1000, FI-02044 Espoo, Finland; heikki.aisala@vtt.fi (H.A.); ulla.holopainen@vtt.fi (U.H.-M.); hanna-leena.alakomi@vtt.fi (H.-L.A.); emilia.nordlund@vtt.fi (E.N.); kaisu.honkapaa@vtt.fi (K.H.)

**Keywords:** high-moisture extrusion cooking, whole fish, underutilised fish, protein fibrillation, meat analogue

## Abstract

Interest in using undervalued forage fish for human consumption has recently increased due to its environmental benefits. However, feasible strategies to process the undervalued fish species to food use are limited. Therefore, this study investigated the possibility to utilise whole (ungutted) Baltic herring as a raw material for hybrid plant-fish meat analogues produced by high-moisture extrusion cooking. The sample properties were compared with ungutted Baltic herring. Produced meat analogues showed sufficiently high microbial quality, with spoilage microbes showing growth levels of under 1.4 log CFU/g. Whole fish and gutted fish extrudates showed uniform flavour- and odour-related sensory profiles. Colour values of the whole fish (L* 57.8) extrudates were similar to the values of gutted fish extrudates (L* 62.0). The whole and gutted fish extrudates had tensile strength in a cross-cut direction of 25.5 and 46.3 kPa, respectively. This correlated with the tearing force of the extrudates analysed by a trained sensory panel. Furthermore, a more explicit protein network was microscopically observed in gutted fish than in whole fish extrudates. The present study showed that high-moisture extrusion cooking enables the use of whole small-sized fish for human consumption.

## 1. Introduction

Although the world is struggling with protein insufficiency and related overfishing [1] resulting from a constantly increasing population, a vast amount of high-quality protein from undervalued fish species remains unexploited for human consumption [2]. Undervalued fish species are far too often used only as animal feed or fertilisers and not directly for human consumption [3]. This is a global issue, as about 20 million tonnes of fish (more than 25% of the whole marine catch) has been annually used for feed, even though 90% of this could be classified as food-grade fish [4]. Various issues affect the acceptance of the fish, including their small size, off-flavours, texture, colour or that they are by-catches of commercially valuable fish species [5]. Examples of particularly small-sized forage fish are blue whiting (*Micromesistius poutassou*), boarfish (*Capros aper*), sprat (*Sprattus sprattus*) and Baltic herring (*Clupea harengus membras*) [3,6]. Regarding Baltic herring, only 3% of the catch in Finland is filleted and utilised as food for the domestic market and 13% for the export market [7]. The rest of the Baltic herring catch in Finland is exploited as fur animal feed, fish meal (including oil) or non-food products for export markets [7]. In the food industry, the utilisation rate of Baltic herring has decreased over the past decades partly due to increased dioxin and polychlorinated biphenyl (PCB) levels in the Baltic Sea [6]. However, during the last 40-year period, toxin levels have decreased up to 80% and are especially low in small-sized fish [8]. Rantakokko et al. [8] showed that the PCB and dioxin levels of Baltic herring <19 cm have decreased under the threshold levels set by the European Union (EU) and are expected to continue decreasing.

Exploiting underutilised fish species for human consumption could contribute to solving the protein needs of the growing population, as fish protein provides a complete amino acid profile and is highly digestible [9]. Additionally, fish are rich in vitamins, minerals and polyunsaturated fatty acids [10]. However, the fish industry lacks economically feasible methods for processing undervalued fish into appealing food products. In particular, filleting of small-sized fish could lead to an unprofitable process if it produces more side-streams than fillets. To overcome this challenge, new techniques and processes need to be developed for utilising the undervalued small-sized fish for human consumption.

Various approaches have been suggested for valorising these undervalued fish species for human consumption, including enzymatic hydrolysis to produce functional and bioactive peptides [3,11] that have also been commercialised as dietary supplements, the use of cross-linking enzymes to produce restructured meat and dry extrusion cooking to produce snacks incorporated with fish [12]. Dry extrusion processing has also been applied to underutilised salmon processing by-products for producing jerky-style salmon products [13].

Although different food processing approaches have already been investigated to valorise underutilised fish species for human consumption, none of these have attempted to use the entire fish in the final product. The aim of this study was to investigate if whole (ungutted) small-sized fish could be used safely in food applications without generating any side-streams. The specific target was to compare the microbiological safety, sensory properties and structure properties of whole fish (ungutted) against gutted fish during high-moisture extrusion cooking with incorporation of plant protein. In high-moisture extrusion cooking, a protein-water blend, with moisture content above 50%, is heated and mechanically sheared inside a barrel, after which the plasticised protein blend enters to a cooling die where the mass is aligned to its final structure. This processing method has been recently re-growing its popularity as a commonly used method for developing plant-based meat alternatives [14]. However, to the best of our knowledge, the only studies that have attempted to texturize fish protein using high-moisture extrusion cooking are from the 1990s or earlier [15,16]. Moreover, these studies focused on creating surimi or fish muscle-based extrudates, whereas the goal of the present work was to use whole fish and thus would avoid the need for extensive pre-processing of fish raw materials.

## 2. Materials and Methods

### 2.1. Raw Materials and Their Preparation

Commercial pea protein isolate (Nutralys F85M) was provided by Roquette (Lestrem, France). Food grade Baltic herring (*Clupea harengus membras*) was provided by Martin Kala Oy with two different gutting degrees: (1) gutted fish (head and intestines removed) and (2) whole fish (ungutted). The fish were caught on 20 May 2019 from the Baltic Sea (Archipelago and Bothnian Sea). The fish were delivered on ice to the laboratory within 24 h of catching. The fish were immediately ground using a meat grinder, homogenised in a kitchen cutter and stored at −20 °C until further processing.

### 2.2. Raw Material Composition

#### 2.2.1. Protein Content

The protein content of the raw materials was measured in triplicate using a Kjeldahl autoanalyser (Foss Tecator Ab, Höganäs, Sweden) according to the AOAC method 2001.11 using a nitrogen conversion factor of 6.25.

#### 2.2.2. Lipid Content

The total lipid content was analysed as the sum of individual fatty acids and was determined in duplicate by modifying the esterification method reported by Glaser et al. [17]. Briefly, 10–20 mg of sample (based on estimated lipid content) was weighed in an autosampler vial, and 20 µg of two internal standards (glyceryl triheptadecanoate and heptadecanoic acid) were added. The triacylglycerols in the sample were esterified by adding 750 µL acidic methanol (with 3 N HCl) and heating for 45 min at 85 °C. The fatty acid methyl esters were extracted by adding 1000 µL of hexane and shaking for 1 min. From the upper hexane layer, 1 µL was injected into an Agilent 7890 gas chromatography (GC) coupled to 5975C mass selective detector (MSD) for gas chromatography-mass spectrometry (GC-MS) analysis. The transport gas was helium with a constant flow rate of 1.2 mL/min. An injector temperature of 240 °C was used in the splitless mode. An HP-FFAP column (25 m × 0.25 mm × 0.33 μm) was used for separating the compounds. The oven temperature was first held at 40 °C for 1.5 min, then increased to 240 °C at a rate of 15 °C/min, and maintained at 240 °C for 9 min. Mass spectrometer (MS) interface temperature of 240 °C was used as well as the quadrupole temperature of 150 °C. The mass spectra were recorded over a 35–600 atomic mass unit range at 3 scans/s.

#### 2.2.3. Moisture and Ash Content

The moisture and ash contents were analysed gravimetrically from the pea protein isolate (R1) and ground fish minces (R2, R3). Additionally, moisture content was analysed from the frozen extrudates (E1–E3). The moisture content was analysed by drying the samples at 105 °C until constant weight. The ash content was analysed after combustion of the samples in a muffle furnace (model N11, Nabertherm GmbH, Lilienthal/Bremen, Germany) at 550 °C for 24 h. Samples were analysed in triplicates.

### 2.3. High-Moisture Extrusion Cooking

#### 2.3.1. Preparation of Extrusion Mixtures

Three different extrusion mixtures, ER1, ER2 and ER3, were prepared with the main component of the recipes comprising pea protein isolate (R1, 99%), gutted fish (R2, 68.4%) and whole fish (R3, 68.4%), respectively. Pea protein isolate (30.6%) was added to the mixtures containing fish in order to reduce the moisture content to a level suitable for high-moisture extrusion cooking (reduction from approximately 80 to 55%, see Table 1). Salt (1%) was incorporated into all mixtures to mimic a possible recipe in food use. The different ingredients were combined and mixed in a kitchen cutter for 60 s in 300 g aliquots.

#### 2.3.2. Extrusion Parameters

**Figure 1:**
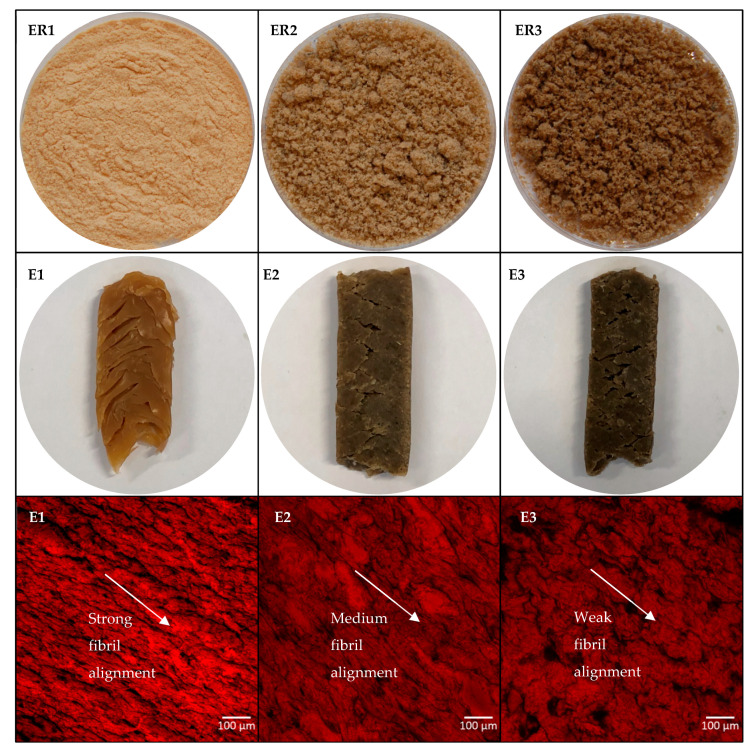
The appearance and the colour of the extrusion raw materials (ER1–ER3) and extrudates (E1–E3), and microstructure of the extrudates (E1–E3). The extrudates were stained with Acid Fuchsin to visualise proteins in red. Fibril alignment direction (strong, medium, weak) is indicated by the arrow.

The prepared mixtures (ER1–ER3) were fed into a lab-scale extruder (Process 11 Hygienic, Thermo Scientific, Karlsruhe, Germany), equipped with a cooling die with dimensions of 4 × 20 × 250 mm (H × W × L), to produce high-moisture extrudates (E1–E3). Before collecting the samples, the optimum extrusion parameters were defined according to the visual appearance of the extrudates by varying the die body (between barrel and cooling die) temperatures from 90 to 140 °C. The target appearance was a fibrous v-shaped structure that resembled a fish backbone alignment (Figure 1, E1–E3). The fibrils were aligned in the same direction as the v-shaped structure. Accordingly, the optimum operational die body temperatures were selected and set as 122, 126 and 126 °C for pea protein, gutted fish and whole fish, respectively. Otherwise, the temperature profile in barrel (from feeder to die body) was the same for all samples: 60–70–80–100–110–150–150 °C. The pea protein control was prepared by feeding flour (300 g/h) and water (333 mL/h) separately, whereas the fish samples were prepared with only flour feed (400 g/h) without any external water feed. Other operational parameters for the three samples were the same (cooling die: 30 °C, screw speed: 200 rpm). Extruder responses (die pressure (bar), torque (Nm) and sample temperature before the cooling die (°C)) were recorded once the extrusion process reached its steady state. The specific mechanical energy (SME) was calculated according to Palanisamy et al. [18].

### 2.4. Determination of Microbial Quality

The microbial quality of the defrosted raw materials was analysed after four months of storage at −20 °C on the same day when producing the extrudates, and the microbial quality of the extrudates was analysed immediately after extrusion cooking. The samples were analysed in triplicate. Ten-gram samples were weighed, diluted with 90 mL of peptone saline and homogenised with a Stomacher (Stomacher 400 Circulator, Seward, Worthing, UK) for 60 s at 260 rpm. A ten-fold dilution series was prepared and plated using the spread plate technique. Plate count agar (PCA, Difco, Bordeaux, France) was used to determine viable counts of aerobic heterotrophic bacteria and spore-forming bacteria (heat treatment 80 °C, 10 min) and incubated at 30 °C for 3 days. *Bacillus cereus* was detected with Mannitol Egg Yolk Polymyxin (MYP) agar (Oxoid, Basingstoke, England) and incubated at 37 °C for 24 h. Hydrogen sulphide-producing bacteria were determined on Lyngby iron agar (with 4% *L*-cysteine supplementation, Oxoid, Basingstoke, England) incubated at 25 °C for 2 days. Enterobacteria were enumerated with Violet Red Bile Glucose Agar (VRBGA; LabM, Heywood, UK,) using the pour plate technique, and coliforms with Chromocult coliform agar (Merck Millipore, Darmstadt, Germany) incubated at 37 °C for 24 h.

### 2.5. Characteristics of the Extrudates

#### 2.5.1. Colour

Colour was measured from the extrusion mixtures (ER1–ER3) and from the extrudates (E1–E3). The colour of the samples was determined by a colorimeter (Minolta Chroma meter, CR-200 Handheld, Osaka, Japan). L* (lightness), a* (green-red) and b* (blue-yellow) values were recorded according to the CIELAB colour space system. The colorimeter was calibrated with a white plate provided by the manufacturer. Ten parallel measurements were conducted for all samples except for sample E1, which was analysed in 7 parallel samples.

#### 2.5.2. Microstructure

The microstructure of the extrudates was analysed by confocal laser scanning microscopy (CLSM) equipment, consisting of a Zeiss LSM 710 (Zeiss, Jena, Germany) attached to a Zeiss Axio Imager. Z2 microscope. The extrudates were cut so as to align the cutting surface parallel to the formed fibrils visible to the eye, i.e., the samples were cut in a 45° angle (along one side of the v-shape in the extrudate) in relation to the longitudinal direction of the extrudate. The protein was stained with Acid Fuchsin to visualise any possible differences in fibril formation by adding 20–50 µL of aqueous 0.1% (*w*/*v*) Acid Fuchsin (BDH Chemicals Ltd., Poole, Dorset, UK) in 1.0% acetic acid onto the cutting surface of the sample. Stained samples were examined on a microscope slide as sealed preparates. HeNe laser line 543 nm was used for excitation of Acid Fuchsin, and the emission was collected at 548–703 nm. Images were assembled from the captured optical sections using a 10× objective (Zeiss EC Plan-Neofluar, numerical aperture of 0.30). The optical sections were taken to a depth of 43–67 μm from the cutting surface of the sample with a z step of 5.1 μm and a resolution of 1024 × 1024 using ZEN software (Zeiss). The final CLSM micrographs, in which the protein appears red, were reconstructed as maximum intensity projections. Representative images of each sample were selected for publication.

#### 2.5.3. Mechanical Properties

The mechanical properties of the extrudates, i.e., tensile strength (kPa) and tearing force (N) values as a function of sample extension (mm), were measured using a Lloyd LS5 material testing machine (Ametek, Berwyn, PA, USA) with a static load cell of 1000 N and a test speed of 2 mm/min. Samples were tempered and measured at 23 °C and 50% relative humidity. The tempered samples were cut into strips of 30 mm in length. The width (15 mm) and thickness (5 mm) values were dictated by the cooling die dimensions. The initial gauge length between the jaws was set to 0.5 mm due to the restricted dimensions of the extrudates. The jaws compressed the samples and caused a negative starting force. Thus, the gauge length value was readjusted manually for each sample to set the starting force to zero. Six parallel samples were used to analyse the mechanical properties of the extrudates.

#### 2.5.4. Cooking Yield and Loss

Cooking yield (%) was measured according to Palanisamy et al. [18] with slight modifications. The thawed extrudates were cut into equal lengths of 3 cm. The samples were heated in water at 80 °C for 20 min. After heating, the samples were drained for 10 min to remove excess water. Cooking yield was calculated according to the following equation:

(1) $$Cooking\;yield=\frac{m_{a}}{m_{b}}\times 100\%\;,$$

where *m_a_* denotes the mass of the extrudate after cooking and *m_b_* the mass before the cooking. Cooking loss (%) was analysed as the dry matter content that dissolved from the extrudate into the cooking water in relation to the initial weight of the sample. The samples were analysed in triplicate.

#### 2.5.5. Free Thiol Groups

The amount of free thiol groups was measured according to Thermo scientific instructions (22582) for Ellman’s reagent (5.5′-dithiobis-(2-nitrobenzoic acid) or DTNB) with slight modifications. The extrudates (E1–E3) were freeze-dried (Alpha 1-4 LSCbasic, Martin Christ, Osterode am Harz, Germany) and ground in a lab-scale ball-mill (MM301 Mixer Mill, Retsch, Haan, Germany) for 2 min at 30 Hz in 50 mL grinding jars with 3 balls. The ground samples (60 mg) were suspended in 1 mL of reaction buffer (0.1 M sodium phosphate buffer, pH 8.0, containing 1 mM Ethylenediaminetetraacetic acid (EDTA)) and mixed thoroughly. Urea (8.0 M) was added to the reaction buffer when indicated. An aliquot of 50 µL of Ellman’s solution (4 mg of DTNB (Sigma-Aldrich, Steinheim, Germany) dissolved in 1 mL of reaction buffer) was mixed with 2.5 mL of the reaction buffer. The suspended protein sample (250 µL) was added to the aliquot and incubated at room temperature for 15 min. The suspension was centrifuged (Centrifuge 5417 R, Eppendorf, Hamburg, Germany) at 10,000× *g* for 10 min, and the absorbance of the supernatant was measured at 412 nm. To quantify the free thiol groups, the absorbance values were plotted against a cysteine standard curve in a concentration range of 0.0–1.5 mM. Three parallel samples were analysed when phosphate buffer without urea was used. When urea was added, two parallel samples were analysed.

### 2.6. Sensory Evaluation

The sensory properties of the three extruded food samples (E1–E3) were studied with generic descriptive analysis by 10 assessors of VTT’s trained food and beverage sensory panel. The frozen food samples were divided into 15 g aliquots and placed in 50 mL closed plastic cups. The aliquots were thawed at ambient temperature for 30 min, warmed up in a 40 °C oven for 15 min and cooled down to ambient temperature for serving. All panellists gave their prior informed consent to participate in the trial. The necessary individual assessor data was collected in accordance with the EU General Data Protection Regulation (GDPR) (2016/679). The protocol for performing the sensory evaluation has been accepted by the Ethical Committee of VTT. The preliminary list of sensory attributes was formulated by 5 sensory experts. This list along with suitable reference products was further refined by all 10 assessors. The reference product intensities were tied to the attributes in a 1 h consensus session. The attribute lists and reference products are presented in the Supplementary Materials in Table S1. After panel training, the assessors evaluated the samples in two duplicate sessions using 0–10 line scales. The samples were presented in a randomised complete block design with three-digit codes. The data were collected using Compusense five version 5.6 (Compusense Inc., Guelph, ON, Canada).

### 2.7. Statistical Analysis

Sensory profiling data were analysed with two-way mixed model analysis of variance (ANOVA) using SPSS version 26 (IBM Corp., Armonk, NY, USA). The panel performance (discrimination, agreement and repeatability) was additionally checked using PanelCheck 1.4.2. One-way ANOVA was used for statistical analysis of the colour, tensile strength, free thiol groups and cooking properties data. F-test or the robust Brown-Forsythe test were used for ANOVA depending on the equivalence of variance, and Tukey’s HSD or Tamhane’s T2 were used as post hoc tests, respectively. The limit for statistical significance was set at *p* < 0.05.

## 3. Results and Discussion

### 3.1. Extruder Responses

The extruder responses pressure, torque and melt temperature (i.e., the sample temperature before the cooling die) were monitored during the extrusion cooking. Differences in extrusion mixtures (ER1 = pea protein isolate, ER2 = gutted fish, ER3 = whole fish) had little effect on the extruder responses. The extrusion pressure was the same for all samples (6 bar). Torque values of 0.7, 0.7 and 0.6 Nm were found for ER1, ER2 and ER3, respectively. The pea control (ER1) and both fish samples (ER2, ER3) showed melt temperatures of 123 and 125 °C, respectively. The calculated SME values of the extrudates were observed to decrease with increasing gutting degree of the fish samples, resulting in values of 83.4, 131.9 and 113.1 kJ/kg for E1, E2 and E3, respectively. The difference in SME values between pea protein and the fish samples can be simply explained by the fact that the feeding rates and feeding style were different, which affected the SME values. However, the lower SME value of E1 in comparison to E2 is related to the lower torque value. Lower torque value indicates that the plasticised protein mass inside the barrel has less resistance towards the shearing due to weaker protein–protein interactions.

### 3.2. Chemical Composition

The chemical composition of the raw materials was measured, whereas the composition of the extrusion mixtures was calculated based on the raw material composition in Table 1. The protein content of the whole fish (R3, 14.2%) was approximately the same as that of the gutted fish (R2, 14.4%). The extrusion mixtures (ER1–ER3) were prepared (see Section 2.3.1) so as to result in similar protein (32.5–34.9%) and moisture (54.3–55.7%) contents for all three samples.

### 3.3. Microbiological Quality

The microbiological quality of the raw materials (R1–R3) and the extrudates (E1–E2) was analysed immediately after extrusion cooking (Table 2). The microbiological quality of the defrosted raw material samples (R1–R3) was generally good, with the number of spore-forming bacteria, *B. cereus* and enterobacteria remaining below the detection limit (<Log 1). However, a low level of H_2_S-producing bacteria was detected in the fish samples (R2, R3). As Lyngby iron agar has been developed specifically for detecting fish spoilage bacteria [19], the H_2_S-producing bacteria were not detected in the pea protein raw material sample (R1), as was expected. During the extrusion cooking process, the heating (set up to 150 °C) efficiently inactivated the microbes and improved the microbiological quality of the extrudates (number of microbes < Log 1). The only exception to this was observed for the gutted fish sample (E2), in which some aerobic heterotrophic bacteria were detected. Only few studies have focused on investigating the microbiological quality of whole fish [20]. Criteria for microbiological quality of foodstuffs has been defined in European Commission legislation, and according to these guidelines, the extrudates had good quality. The present results indicate that small-sized springtime Baltic herring could offer a suitable raw material for extruded ready-to-eat products.

### 3.4. Colour

The extrusion mixtures and extrudates were analysed for colour (Table 3) and visual appearance (Figure 1). Extrusion cooking was found to have a browning effect on the samples, which presumably occurred due to the Maillard reaction. Although the colour detected in the mixtures (ER1–ER3) was lighter than that of the extrudates (E1–E3), the difference in the lightness between the three samples was higher before extrusion (L* 65.0–97.7) than after extrusion (L* 57.8–64.7). In general, the colours of the extrudates were similar to those reported for high-moisture soy-gluten extrudates (L* 58.0–61.4) by Chiang et al. [21]. In the present study, the samples with whole fish were observed to be darker than the gutted fish sample (Table 3 and Figure 1), most likely due to the darker colour of the viscera and head included in sample E3. This explanation would be in line with the study of Sathivel et al. [22], who reported that herring viscera and head hydrolysates (L* 79.3 and 74.6, respectively) had darker colour than herring body hydrolysate (L* = 84.3, viscera and head removed). However, they also reported lighter colour for whole-herring hydrolysate (L* = 89.4) than for the body hydrolysate, which is the opposite of what was found in the present study regarding the Baltic herring extrudates and extrudate mixtures.

### 3.5. Structure Properties

The fibril alignment of the proteins in the extrudates (E1–E3) was illustrated in microscopy images (Figure 1) by staining the protein with Acid Fuchsin. The pea protein extrudate (E1) had strong fibril alignment with clear arrangement of the proteins along the same longitudinal direction, similar to what was visualised by Chiang et al. [21] for mixed soy protein and gluten-based meat analogues, as well as by Palanisamy et al. [18] for lupin protein extrudates under scanning electron microscope. A medium level of fibril alignment was observed in the gutted fish sample (E2), though some proteins appeared in round clusters. The fibril alignment was clearly lower in the whole fish sample (E3) with weaker micro-level protein arrangement than in samples E1 and E2.

**Figure 2:**
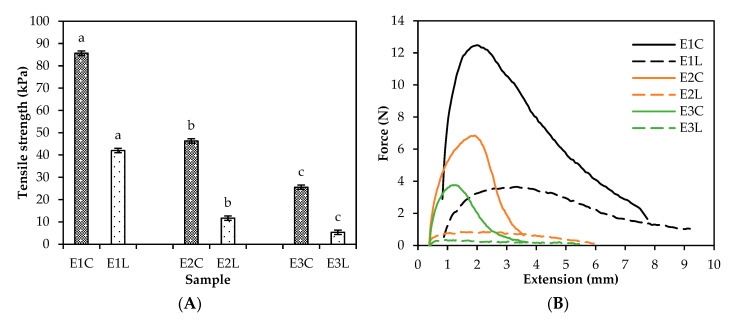
Longitudinal (L) and cross-directional (C) (**A**) tensile strength (kPa) of the extrudates (E1–E3), (**B**) tearing force (N) of the extrudates (E1–E3) as a function of extension (mm). Different letters (a, b, c) indicate the significant differences (*p* < 0.05) within sample groups ER1–ER3 and E1–E3.

Tensile strength, i.e., the maximum stress during the pulling of the extrudate before breaking, was measured along both the longitudinal and cross-sectional directions (Figure 2A). The ideal conditions would be when the strength is higher in one direction and weaker in the other, similar to meat fibres [23]. In this study, a clear trend was observed showing that the cross-directional strength was higher than the longitudinal strength (Figure 2A). When the tearing force acting upon the extrudate is plotted against its extension (Figure 2B), the cross-sectional forces had an apparent maximum peak, whereas the forces in the longitudinal direction were similar throughout the extension. Thus, it can be concluded that all the samples resist tearing in the cross-sectional direction but are easy to break in the longitudinal direction. Similar results were observed in the study of Chiang et al. [21], which found that soy- and gluten-based meat analogues had stronger structure in one direction than in the other, similar to chicken breast that was used as the reference sample. In this study, a clear trend was observed of decreasing tensile strength as one moves from the pea protein sample (E1) to the gutted fish (E2) and whole fish samples (E3). Pea protein isolate control (E1) had the highest tensile strength of 85.7 and 42.0 kPa in cross and longitudinal direction, respectively. The tensile strength of the gutted fish extrudate (E2) was nearly half kPa units weaker in the cross-direction (46.3 kPa) and even weaker in the longitudinal direction (11.7 kPa). Tensile strength decreased further in the whole fish extrudate (E3) resulting in cross and longitudinal strengths of 25.5 and 5.3 kPa, respectively. These results were in line with the trend observed in microscopy, where the pea protein sample (E1) had the strongest fibril alignment and the whole fish sample (E3) had the weakest.

Extrusion cooking temperature is an important parameter that affects the structure formation, as was seen in preliminary trials (data not shown) in which the fibril formation of a freeze-dried fish mince (no addition of pea protein) took place at a higher melt temperature (153 °C) than was studied for the extrudates E1–E3 with pea protein (123–125 °C). This would explain the weaker structure of the fish samples (E2–E3), since the fibril formation at 125 °C is mainly resulted from the pea protein isolate. Thus, with higher temperatures, the fibril alignment of the fish samples could be improved. In addition, also, the pea protein forms a stronger network at higher temperatures [24], which would in turn help to improve the structure.

To get further understanding on the fibril formation during extrusion, the thiol groups were measured to have an indication of the extent of disulphide bond formation in pea and fish samples during extrusion cooking (Table 3). It is widely known that the formation of disulphide bonds between protein molecules contributes to polymerisation, and thus, to the functionality of the proteins. For instance, Osen et al. [25] investigated the role of disulphide bond formation during the high-moisture extrusion cooking, and they concluded that the disulphide bond formation has a major role in structure formation. In the present study, thiol groups were measured with two different reaction buffers: sodium phosphate buffer with and without urea. Urea was added to reduce the non-covalent bonds, thus making the sample more soluble and the thiol groups more available for the Ellman’s reagent to react in the analysis.

The amount of free thiol groups without reducing the non-covalent bonds was 0.6, 6.2 and 3.2 mmol/mg before extrusion and 3.2, 5.1 and 2.5 mmol/mg after the extrusion for E1, E2 and E3, respectively (Table 3). When the non-covalent bonds were reduced, the amount of free thiol groups was 0.9, 7.0 and 3.4 mmol/mg before extrusion and 3.6, 7.8 and 5.1 mmol/mg after the extrusion for E1, E2, and E3, respectively. Therefore, it was observed that the gutted fish samples (ER2, E2) had higher amounts of free thiol groups in comparison to the whole fish (ER3, E3) before and after extrusion cooking with and without reduction of non-covalent bonds in the thiol group analysis. Cysteine is usually absent in fish collagen leading to a conclusion that it is not able to form disulphide bonds, as was observed in the studies of Kittiphattanabawon et al., Muyonga et al. and Montero et al. [26,27,28]. This would explain the lower amount of free thiol groups observed in the current study in whole fish (includes head that contains collagen) than in the gutted fish (no head, less collagen) samples. Benjakul and Morrissey [29] reported that cysteine concentration was lower in the Pacific whiting solids waste (heads, viscera and muscle tissue) than in the muscle tissue alone, which is in line with the difference in free thiol groups observed in the present study for the gutted and whole fish. This can also explain the weakest structure of whole fish extrudates observed in the tensile strength and microscopy analyses.

The amount of free thiol groups in the unreduced pea protein sample increased after extrusion, whereas in fish samples it decreased, although fish samples showed weaker structure formation than pea protein samples. This was in contrast with what was expected, as the decrease in free thiol groups indicates for disulphide bond formation, which is important in structure formation. Isolation of plant protein, including pea protein, often requires wet-processing with pH shifting and possible heating steps, which alters the native state of the protein [24]. When the protein is treated in such conditions, the protein coil starts to open up, leading to an exposure of reactive groups, including thiol groups [30]. These proteins start to aggregate when the exposed groups react with each other, leading to a compact structure with thiol groups buried inside the protein aggregates, leading to a low concentration of detectable free thiol groups. Therefore, more thiol groups where revealed when extrusion cooking unfolded the aggregated proteins and rearranged them into more organised network (as was observed in microscopy images in Figure 1). When the non-covalent bonds of the samples were reduced, the amount of free thiol groups increased in all samples after extrusion cooking. This showed that the free thiol groups were buried inside the protein structure formed by non-covalent bonds and were unavailable for the analysis by Ellman’s reagent.

### 3.6. Sensory Profile

**Figure 3:**
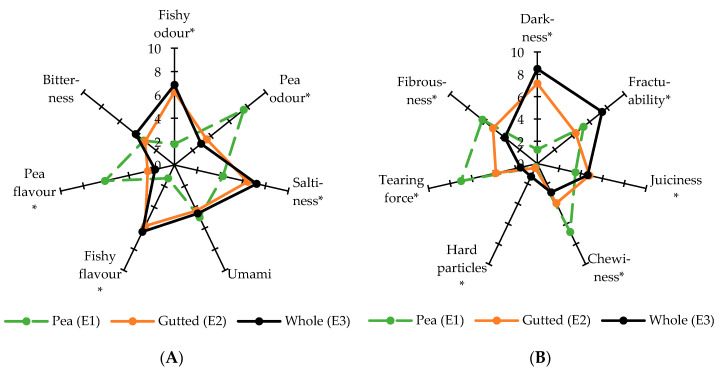
Descriptive sensory profiles of the extrudates: (**A**) the taste- and odour-related profiles, (**B**) the structure and appearance related profiles. The attributes marked with an asterisk (*) have statistically significant differences between samples (*p* < 0.05 in the two-way mixed model analysis of variance (ANOVA)).

The sensory panel evaluated the sensory profiles of the three extrudate samples (E1–E3) on their odour, appearance, texture, taste, flavour, and mouthfeel properties using 14 different sensory attributes. These three extrudates had statistically significant differences in all other attributes apart from umami and bitterness (Figure 3, Supplementary Table S2). The sensory panel had a good agreement on the values of sensory attributes, was able to discriminate between the samples and had a good repeatability between evaluation sessions as demonstrated by close clustering of assessors in Tucker-1 plots, and by individual assessors having *p* < 0.2 and MSE < 2 (mean squared error) in most sensory attributes.

The two fish samples, E2 (gutted) and E3 (whole), differed from each other mainly in terms of structure attributes (Figure 3B). The whole fish was perceived darker, having more hard particles and being more fragile, whereas the gutted fish was perceived chewier, more fibrous and having a higher tearing force. Fibrousness, tearing force and chewiness were perceived the most intense in pea protein extrudate (E1). Both fish samples were perceived juicier than the pea protein samples. These findings were in line with the trend observed in the instrumental texture and microscopy analysis where the pea protein extrudate (E1) had the strongest structure and the whole fish extrudate (E3) had the weakest.

On the other hand, the two fish samples were similar to each other in terms of odour and flavour (Figure 3A). This was in contrast to the pea protein sample, which differed from the fish samples in terms of fishy odour, pea odour, saltiness, fishy flavour and pea flavour. Naturally, the fishy odour and flavour were present in fish samples and the pea odour and flavour in pea samples. The fish samples were perceived saltier than the pea protein samples.

Studies on sensorial properties of whole fish or fish products in comparison to gutted fish are limited. In the study of Cakli et al. [20], the sensory quality of whole and gutted sea bream and sea bass were compared during 14 days of storage. However, they used a quality grading method instead of the descriptive sensory profiling. Surasani [31] reviewed several sensory-related studies on fish meat-based extrudates. These experimental set-ups were also different from the present study, since they examined the acceptability of the products rather than studying the descriptive sensory profile of the products.

### 3.7. Cooking Properties

The cooking yield and loss of the extrudates were analysed to illustrate the strength of the samples during meal preparation. Furthermore, the cooking yield is an important parameter when considering the sensorial properties of the product, such as juiciness [32]. Moisture contents of the extrudates were measured before and after cooking to verify the cooking yield results. In general, the cooking loss, i.e., the amount of dry matter dissolved into the cooking water during boiling, was low (≤5.8%). The cooking loss of the whole fish extrudate (E3, 5.8%) was significantly higher in comparison to the gutted fish (E2, 2.9%) and pea protein extrudate (E1, 3.0%). The highest cooking yield was observed for the whole fish extrudate (E3, 183.0%), which also had significantly higher moisture content after boiling (77.5%) in comparison to the gutted fish (E2, 70.2%) and pea protein extrudate (E3, 70.3%). The cooking yield of the gutted fish extrudate (E2, 137.8%) was the lowest, followed by the pea protein extrudate (E1, 148.2%). Regardless of the differences in cooking yield, the moisture contents of E1 and E2 samples were almost the same after boiling. The higher cooking yield and moisture content of the whole fish sample could be explained by its fractured structure, having more space available for water-intake inside the protein network. A similar observation was made by Palanisamy et al. [33] when they studied the influence of iota carrageenan addition to the properties of soy protein meat analogues. They claimed that the cooking yield was reduced by carrageenan addition, which led to a more compact protein network making the water penetration between the proteins more difficult. The cooking yield values reported by Palanisamy et al. [33] were similar (138.2–145.2%) to the values of E1 and E2 samples in the present study.

## 4. Conclusions

High-moisture extrusion cooking provides a potential solution for efficient raw material use for underutilised fish species by enabling the use of small-sized whole fish, thus avoiding the need for laborious gutting step of the fish. The microbiological quality was similar in all extrudates made from whole fish, gutted fish or pea protein isolate, indicating that sufficient microbiological quality can be achieved for ready-to-eat products made from entire fish when using the high-moisture extrusion cooking process. However, the stability of the microbial quality during storage as well as oxidation stability needs further investigation. Moreover, the flavour and odour properties of the whole and gutted fish were very similar. Yet, the structure of the whole fish extrudate was weaker than the gutted fish and pea protein isolate, which suggests the need for adjusting the operational parameters of the extrusion process. Although this concept needs further optimisation, the results indicate its clear potential as an alternative for conventional minced meat or fish meat products. Furthermore, the process is expected to be suitable also for small-sized and underutilised fish species other than Baltic herring. Further studies are needed on scale-up process and consumer acceptance.

## Figures and Tables

**Table 1:** Measured chemical composition of the raw materials (R1–R3) and calculated composition of the extrusion mixtures (ER1–ER3) (as is, %), where 1 indicates pea protein, 2 gutted fish and 3 whole fish.

Sample	Protein	Fat	Ash	Moisture
R1	74.4 ± 0.0	5.3 ± 0.1	3.7 ± 0.5	6.6 ± 0.0
R2	14.4 ± 1.6	2.3 ± 0.1	1.5 ± 0.0	81.8 ± 0.1
R3	14.2 ± 0.4	2.2 ± 0.1	2.2 ± 0.1	80.3 ± 0.2
ER1 *	34.9	2.5	1.6	55.7
ER2 *	32.9	3.2	2.1	54.3
ER3 *	32.5	3.1	2.6	54.3

* = calculated measures based on the results of R1–R3.

**Table 2:** Microbiological quality of the raw materials (R1–R3) and extruded food samples (E1–E3) presented as Log colony forming units per gram.

Microbial Groups Analyzed	Raw Materials			Extrudates		
	R1	R2	R3	E1	E2	E3
Aerobic heterotrophic bacteria	2.4 ± 0.7	2.1 ± 0.1	3.0 ± 0.4	<1	1.4 ± 0.4	<1
Spore-forming bacteria	<1	<1	<1	<1	<1	<1
*Bacillus cereus*	<1	<1	<1	<1	<1	<1
H_2_S-producing bacteria	<1	1.3 ± 0.3	1.3 ± 0.6	<1	<1	<1
Enterobacteria	<1	<1	<1	<1	<1	<1
Coliformic bacteria	<2	<2	<2	<1	<1	<1

**Table 3:** Colour values (L*, a*, b*), free thiol groups (mmol/mg) analysed in sodium phosphate buffer with (NaP + U) or without (NaP) urea and cooking properties (before and after cooking) of the extrusion mixtures (ER1–ER3) and extrudates (E1–E3), where 1 indicates pea protein, 2 gutted fish and 3 whole fish. Different letters (a, b, c) indicate the significant differences (*p* < 0.05) within sample groups ER1–ER3 and E1–E3.

Sample	ER1	ER2	ER3	E1	E2	E3
L* (lightness)	97.7 ± 0.1 ^a^	75.3 ± 0.5 ^b^	65.0 ± 0.2 ^c^	64.7 ± 1.1 ^a^	62.0 ± 2.2 ^b^	57.8 ± 1.6 ^c^
a* (red/+, green/−)	0.0 ± 0.0 ^a^	−1.5 ± 0.1 ^c^	−0.6 ± 0.2 ^b^	4.1 ± 0.2 ^a^	−0.4 ± 0.1 ^b^	0.4 ± 0.1 ^c^
b* (yellow/+, blue/−)	2.2 ± 0.1 ^a^	−1.1 ± 0.3 ^b^	−2.9 ± 0.2 ^c^	3.0 ± 1.1 ^a^	−5.9 ± 1.9 ^b^	−6.2 ± 1.4 ^b^
Thiols, NaP	0.6 ± 0.1 ^c^	6.2 ± 0.2 ^a^	3.2 ± 0.2 ^b^	3.2 ± 0.0 ^b^	5.1 ± 0.6 ^a^	2.5 ± 0.0 ^b^
Thiols, NaP + U	0.9 ± 0.0 ^c^	7.0 ± 0.0 ^a^	3.4 ± 0.0 ^b^	3.6 ± 0.5 ^a^	7.8 ± 1.2 ^a^	5.1 ± 0.3 ^a^
Moisture before (%)	-	-	-	54.2 ± 0.6 ^b^	56.3 ± 0.2 ^a^	54.9 ± 1.2 ^a,b^
Moisture after (%)	-	-	-	70.3 ± 0.1 ^b^	70.2 ± 0.4 ^b^	77.5 ± 0.9 ^a^
Cooking loss (%)	-	-	-	3.0 ± 0.1 ^b^	2.9 ± 0.2 ^b^	5.8 ± 0.3 ^a^
Cooking yield (%)	-	-	-	148.2 ± 1.1 ^b^	137.8 ± 1.1 ^c^	183.0 ± 4.0 ^a^

## Supplementary Materials

The following are available online at https://www.mdpi.com/2304-8158/9/11/1541/s1. Table S1: Sensory attributes, attribute descriptions and reference products with their bound intensities in the generic descriptive analysis. The sample attributes were evaluated in the shown order, Table S2: Mean intensities (n = 2 × 10) and standard deviations of sensory attributes in the sensory profile of the three extruded food samples. Significant differences between samples are based on a univariate two-way mixed model ANOVA. Partial η2 is an estimate of effect size.
