# Supplementary material for: Comparison of Whole and Gutted Baltic Herring as a Raw Material for Restructured Fish Product Produced by High-Moisture Extrusion Cooking
Source: Foods. 2020 Oct 26;9(11):1541. doi: 10.3390/foods9111541 (PMC7692608; doi:10.3390/foods9111541)
Supplement: Supplementary file 1 [file foods-09-01541-s001.pdf]

Supplementary data

Table S1. Sensory attributes, attribute descriptions and reference products with their bound intensities in the generic descriptive analysis. The sample attributes were evaluated in the shown order.

Attribute	***Description***	***Reference product***	***Intensity*** [a]
Odour			
Fishy odour	Fish-like odour, reminiscent of herring, herring skin or fish bones	Herring filet (AB Salmonfarm Oy, Kasnäs, Finland) fried to 90 °C in rapeseed oil in a saucepan	8
Pea odour	Odour reminiscent of dried peas or beans	10 g of pea protein isolate suspension (1:7 protein: water; Nutralys F85M, Roquette, France)	8
Appearance and texture			
Sample darkness	Sample darkness ranging from light (caramel-like) to dark (rye bread or cooked liver like)	None	
Sample fracturing	The tendency of the sample to have cross-sectional streaks and cracks	Reference pictures of wet-extruded samples: Pea protein concentrate (55% protein, Vestkorn, Norway)	0 and 3
		Faba protein concentrate (65% protein, Vestkorn, Norway)	7
		Rice protein, Remypro80N+ (Beneo, Belgium)	9
Taste and flavour			
Saltiness	Salty taste	0.5% NaCl solution (Meira Oy, Helsinki, Finland)	8
Umami	Umami taste	Solution containing 0.025% MSG and 0.02% GMP/IMP mixture (Ajinomoto Foods Europe SAS, Germany)	7
Fish flavour	Fish-like flavour	Fried herring filet (same as fishy odour)	6
Pea flavour	Pea-like flavour	Pea protein isolate (same as pea odour)	6
Bitterness	Bitter taste that becomes evident at the end of chewing	Pea protein isolate (same as pea odour)	9
Mouthfeel			
Juiciness	Level of moisture that is sensed when pressing the sample with a tongue and chewing		8
Chewiness	The amount of chewing force required to bite the sample in half	None	
Hard particles	Number of hard part particles felt during chewing of the sample	None	
Tearing force	Amount of force needed longitudinally to tear the sample in two pieces	None	
Fibres	Number of meat-like fibres after twisting the sample between fingers	Picture set of wet-extruded samples: Pea protein concentrate (55% protein), Vestkorn, Norway	0
		Pea protein isolate Nutralys F85M (Roquette, France)	8
		Vital wheat gluten (Beneo, Germany)	10

[a] The attribute intensities were measured with 0-10 line scales, where 0 = the attribute was not perceived, 10 = the attribute was very intense. The reference products were bound to these intensities to reduce scale use differences among the panel.

Table S2. Mean intensities (n=2×10) and standard deviations of sensory attributes in the sensory profile of the three extruded food samples. Significant differences between samples are based on a univariate two-way mixed model ANOVA. Partial η^2 is an estimate of effect size.

Attribute	F test p value	Product partial η^2	E1			E2			E3		
Fishy odour	<0.001	0.78	1.8	(2.6)	c	6.3	(1.4)	b	6.9	(1.2)	a
Pea odour	<0.001	0.78	7.6	(1.0)	a	3.5	(2.4)	b	2.9	(2.4)	c
Darkness	<0.001	0.97	1.3	(1.1)	c	7.2	(0.7)	b	8.5	(0.6)	a
Fracturing	0.001	0.57	5.3	(1.9)	b	4.4	(1.3)	c	7.4	(0.9)	a
Saltiness	<0.001	0.83	4.3	(1.5)	c	6.4	(1.3)	b	7.2	(0.7)	a
Umami	0.390 †	0.10	5.0	(1.4)		4.3	(1.9)		4.6	(1.9)	
Fishy flavour	<0.001	0.83	1.3	(1.7)	c	5.8	(1.3)	b	6.3	(1.1)	a
Pea flavour	<0.001	0.82	6.1	(1.4)	a	2.3	(1.5)	b	1.7	(1.0)	c
Bitterness	0.165 †	0.18	3.4	(1.7)		3.2	(1.7)		4.3	(2.3)	
Juiciness	0.019	0.36	3.5	(1.6)	b	4.8	(1.5)	a	4.6	(1.8)	a
Chewiness	<0.001	0.93	6.8	(1.3)	a	3.9	(1.5)	b	2.9	(1.5)	c
Hard particles	0.010	0.40	0.3	(0.6)	b	0.4	(0.6)	b	1.3	(1.2)	a
Tearing force	<0.001	0.91	7.0	(1.3)	a	3.8	(1.5)	b	1.6	(1.1)	c
Fibres	0.037	0.31	6.3	(2.4)	a	5.1	(1.5)	b	3.7	(1.6)	c

() = standard deviation, a-c = the significant differences between samples in each attribute (Tukey HSD test, p<0.05)
† = non-significant ANOVA, no post hoc performed, E1 = pea protein isolate control, E2 = gutted fish, E3 = whole fish
